# A self‐assembled, genetically engineered, irradiated tumor cell debris vaccine

**DOI:** 10.1002/EXP.20220170

**Published:** 2024-03-06

**Authors:** Yajie Sun, Yan Hu, Yuanyuan Geng, Chao Wan, Yang Liu, Yifei Liao, Xiujuan Shi, Jonathan F. Lovell, Kunyu Yang, Honglin Jin

**Affiliations:** ^1^ Cancer Center Union Hospital Tongji Medical College Huazhong University of Science and Technology Wuhan China; ^2^ Hubei Key Laboratory of Precision Radiation Oncology, Union Hospital Tongji Medical College, Huazhong University of Science and Technology Wuhan China; ^3^ College of Biomedicine and Health and College of Life Science and Technology Huazhong Agricultural University Wuhan China; ^4^ Key Laboratory of Polymer Ecomaterials Changchun Institute of Applied Chemistry Chinese Academy of Sciences Changchun China; ^5^ Division of Infectious Diseases Department of Medicine Brigham and Women's Hospital Harvard Medical School Boston Massachusetts USA; ^6^ Department of Chemical and Biological Engineering State University of New York University at Buffalo Buffalo New York USA

**Keywords:** anti‐PD‐1, cancer immunotherapy, cancer vaccine, genetically modified tumor membrane, hydrogel, lung cancer, radiated tumor cell debris

## Abstract

Vaccine‐based therapeutics for cancers face several challenges including lack of immunogenicity and tumor escape pathways for single antigen targets. It has been reported that radiotherapy has an in situ vaccine effect that provides tumor antigens following irradiation, helping to activate antigen‐presenting cells (APCs). Herein, a new vaccine approach is developed by combining genetically engineered irradiated tumor cell debris (RTD) and hyaluronic acid (HA), termed HA@RTD. A cancer cell line is developed that overexpresses granulocyte‐macrophage colony‐stimulating factor (GM‐CSF). A hydrogel was developed by covalent conjugation of HA with RTD proteins that acted as a potent vaccine system, the effects which were probed with T cell receptor sequencing. The engineered vaccine activated antitumor immunity responses and prevented tumor growth in mice even with a single immunization. HA@RTD vaccine efficacy was also assessed in therapeutic settings with established tumors and in combination with immune checkpoint blockade.

## Graphical abstract

Sun et al. report on a personized cancer vaccine generated by irradiating GM‐CSF overexpressed tumor cells (providing antigens and stimulation signal for APCs), covalent conjugation with hyaluronic acid (improving the persistence of antigen stimulation). The engineered vaccine activated antitumor immunity responses and prevented tumor growth in mice with a single immunization.

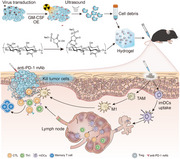

## 1 INTRODUCTION

Lung cancer is considered as one of the malignant tumors with the greatest impact.^[^ ^1^, ^2^ ^]^ The main treatment methods for lung cancer including surgery, radiotherapy, chemotherapy, targeted therapy, and immunotherapy, while the overall therapeutic effect in late‐stage disease is limited and drug resistance is prone to occur.^[^ ^3^ ^]^ In past years, tumor vaccines targeting tumor associated antigens or neoantigens to induce specific immune response, emerged as a promising field of active immunotherapy.^[^ ^4^, ^5^, ^6^ ^]^ The success of tumor vaccines largely depends on the effectiveness of tumor antigens, including complexity and duration, and stimulatory signals to antigen‐presenting cells (APCs). Activated APCs effectively induces the activation and proliferation of anti‐tumor T cells in lymph nodes or other peripheral immune organs where they later infiltrate into the tumor microenvironment (TME).^[^ ^7^, ^8^ ^]^

At present, the experimental lung cancer vaccine Tedopi is a vaccine composed of optimized epitopes from five tumor‐associated antigens. Compared with mucinous glycoprotein vaccines, epidermal growth factor vaccines or ganglioside vaccines that modify only one tumor‐associated antigen, Tedopi vaccine led a significantly longer survival,^[^ ^9^ ^]^ indicating that it is important to construct vaccines with a broad antigen spectrum for anti‐tumor immunity. Due to the heterogeneity of tumor cells, it is difficult for a single antigen to generate sufficient T cells against all tumor cells, which severely limits the efficacy of tumor vaccines.^[^ ^10^ ^]^ Radiation has been demonstrated to induce immunological impact in vivo.^[^ ^11^, ^12^ ^]^ Radiation causes double strand DNA damage and increases the tumor mutational load, where the novel mutations may serve as effective tumor neoantigens.^[^ ^13^, ^14^ ^]^ It has been reported that radiation can induce new antigenic targets, which may explain the in situ vaccine effect of radiation and synergy between radiation and immunotherapy.^[^ ^15^ ^]^ Radiation can also induce damage‐associated molecular patterns (DAMP), upregulate the major histocompatibility complex class I (MHC‐I), and activate the cGAS‐STING pathway to promote the release of type I interferon, which enhances the antigen presenting and leads to increasement of anti‐tumor CD8^+^ T cells,^[^ ^16^, ^17^, ^18^ ^]^ functioning as an in situ tumor vaccine which would inhibit tumor growth both locally and systemically. Therefore, radiated tumor cell debris may contain a broad antigen spectrum for anti‐tumor immunity and is ideal material for tumor vaccine. Another important factor restricting the effect of tumor vaccine is the exposure duration of tumor antigens to immune cells, where the prolonged stimulation time on immune cells helps to amplify the immunological responses.^[^ ^19^, ^20^ ^]^ Hyaluronic acid (HA) is the most common macromolecule in the connective tissues of vertebrates, behaving as linear polysaccharide composed of glucuronic acid and N‐acetylglucosamine repeats.^[^ ^21^ ^]^ It has a wide range of applications because of its biodegradability and biocompatibility.^[^ ^22^, ^23^ ^]^ Activated HA that was modified via the esterification reaction between *N*‐hydroxysuccinimide (NHS) and the carboxyl groups of HA could interact with proteins and assemble into hydrogel.^[^ ^24^ ^]^ In addition, stimulatory signals, such as granulocyte‐macrophage colony‐stimulating factor (GM‐CSF), activate the antigen‐presenting dendritic cells (DCs) that further benefit the anti‐tumor immune responses.^[^ ^25^, ^26^, ^27^ ^]^

In this study, we constructed a novel tumor vaccine, combing the GM‐CSF expressing irradiated tumor cell debris (RTD) and HA to form a hydrogel, where GM‐CSF was overexpressed on tumor cell membrane by adding transmembrane peptide with GM‐CSF. Irradiated lung cancer cells debris is composed of a broad range of tumor antigens and GM‐CSF activates DCs to present the antigen to T cells, which further induce the activation of T cells. The hydrogel form improves the persistence of antigens to induce a more durable immune response. Taken together, our data showed that this formulation promoted innate and adaptive immunity activation, and inhibited tumor growth by just one injection.

## 2 RESULTS AND DISCUSSION

### 2.1 Construction and characterization of the HA@RTD hydrogel vaccine

**FIGURE 1:**
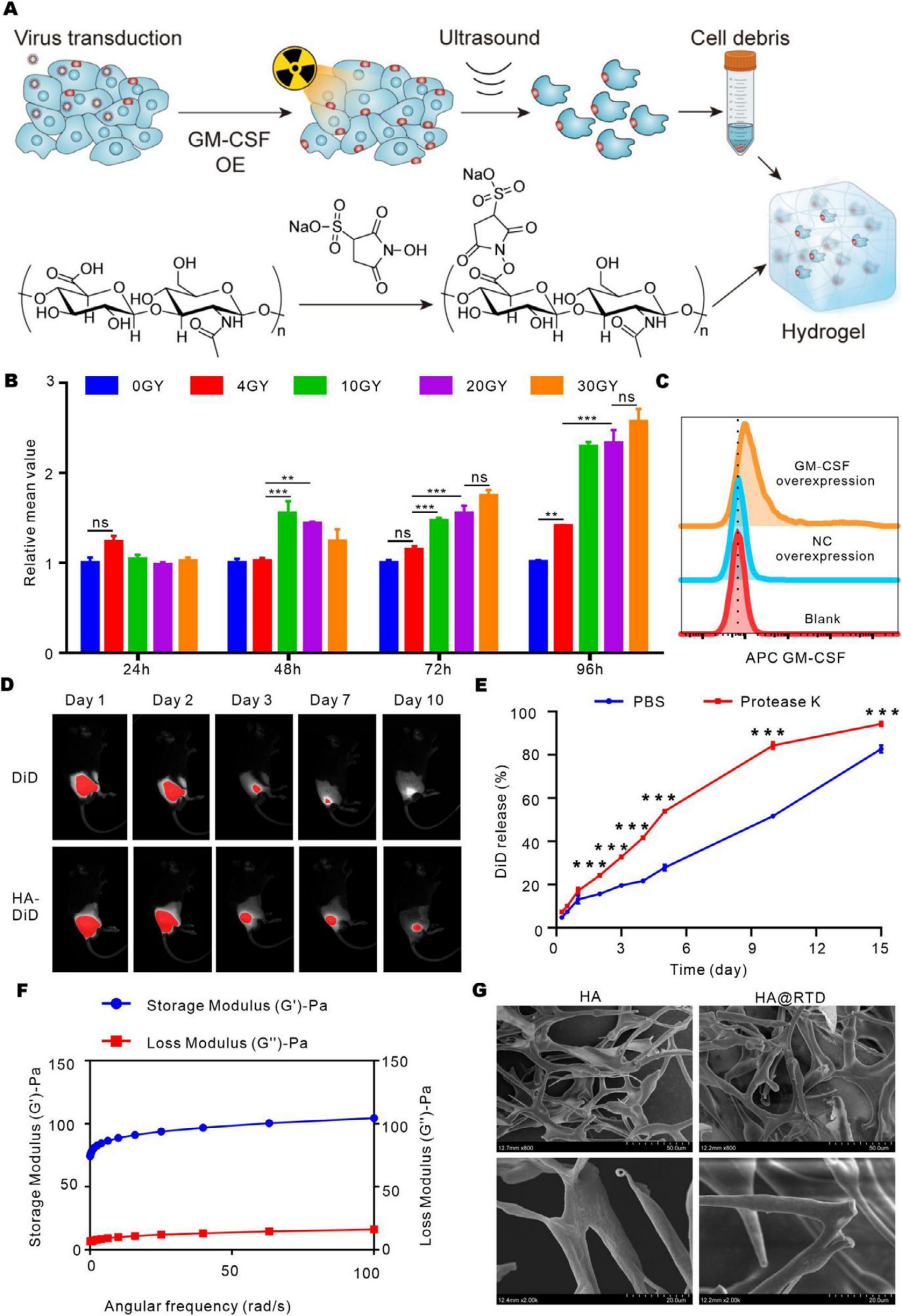
Characterization of the HA and HA@RTD hydrogel vaccine. (A) Schematic of the tumor vaccine design and the potential function model. (B) Flow cytometry analysis of CRT expression on Lewis cells after radiation at different time points. (C) Flow cytometry analysis of GM‐CSF expression on surface of Lewis cells. (D) The DiD imaging of the distribution of the HA‐DiD and free DiD at indicated time points in vivo. (E) Comparison of the release rate of DiD from the HA in PBS or proteinase K solution. (F) Step‐strain time‐dependent rheological analysis of the HA@RTD. (G) Scanning electron microscope (SEM) images of HA and HA@RTD.

Radiation can kill tumor cells and induce immunologic cell death to activate anti‐tumor immune responses. To further improve the immunological effect, tumor cells were modified by overexpressing transmembrane GM‐CSF. The mixture of activated hyaluronic acid (HA‐NHS) and radiated tumor cell debris are capable of self‐assembly into hydrogel, termed HA@RTD hydrogel (Figure 1A).^[^ ^28^, ^29^ ^]^

To optimize irradiation dosing and timing for better immunogenicity of irradiated tumor cells, we measured the expression of calreticulin (CRT) by flow cytometry after treating with different dose of radiation and time. We chose 20 gray radiation and 96 h for later experiments (Figure 1B). The flow cytometry data and western blot data also showed the overexpression of GM‐CSF on the membrane of Lewis cells (Figure 1C and Figure S1A). Additionally, we subcutaneously injected HA‐DiD and free DiD in mice and detected the signal by the ChemiDoc MP Imaging System. As shown in Figure 1D, the DiD signal disappeared at day 10 in free DID group, while it was still detectable in HA‐DiD group. We also used hydrosoluble dye Cy5 to test the releasing in vivo. The Cy5 signal in HA@RTD‐Cy5 group disappeared after day 7, while the Cy5 signal in RTD‐Cy5 disappeared at day 3 (Figure S1B). To further study the release rate of HA hydrogel in vitro, we measured the release of DiD from HA‐DiD in phosphate buffered saline (PBS) or proteinase K. As shown in Figure 1E, proteinase K significantly increased the release rate of DiD from HA. We tested the size distribution of the irradiated tumor cell debris by nanoparticle tracking analysis, which showed that the size varied from 100 to 10000 nm (Figure S1C). We also evaluated the rheology properties of HA@RTD hydrogel by the rheometer. When the strain constant is kept at 0.1%, the storage modulus (*G*‘) and loss modulus (*G*’’) of the hydrogel slightly depends on the angular frequency (0.1–100 rad s^−1^), characterizing the stiffness and stability of the hydrogel (Figure 1F). In addition, the structure of the HA@RTD hydrogel was analyzed by cryo‐scanning electron microscope (cryo‐SEM). As shown in Figure 1G, HA and HA@RTD hydrogel was assembled into fiber network. Taken together, these data indicated that we successfully developed a sustained‐releasable and tumor cell debris‐loaded hydrogel.

### 2.2 In vitro activation of dendritic cells and macrophages

**FIGURE 2:**
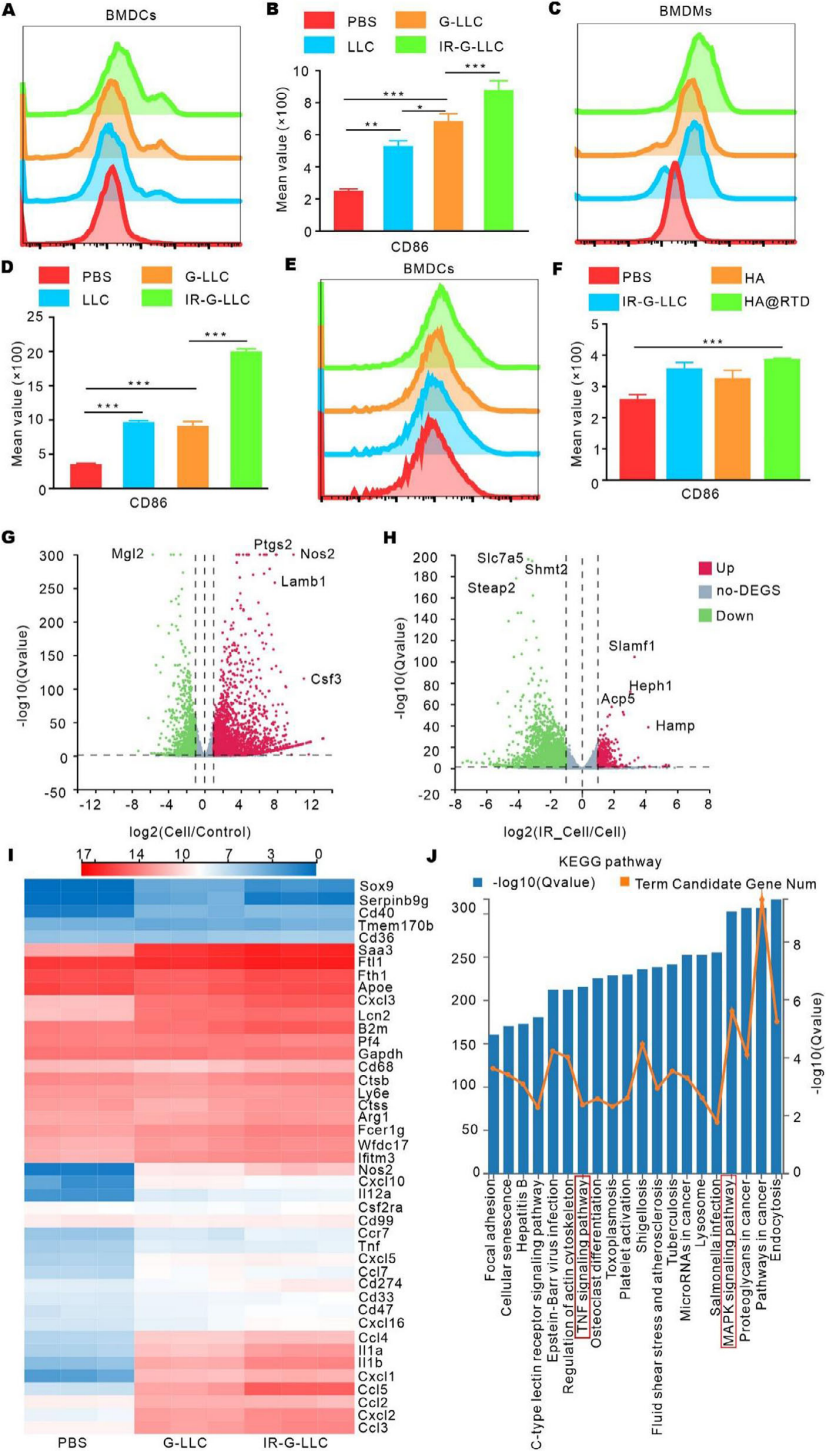
Irradiated tumor cell debris activated DCs and macrophages in vitro. Flow cytometry analysis of CD86 expression in (A,B) BMDCs and (C,D) BMDMs treated with PBS, Lewis cell debris (LLC), overexpression GM‐CSF Lewis cell debris (G‐LLC), and irradiated overexpression GM‐CSF Lewis cell debris (IR‐G‐LLC). (E,F) Flow cytometry analysis of CD86 expression in BMDCs treated with PBS, IR‐G‐LLC, HA, or HA@RTD hydrogel. (G) The volcano plot showing the differentially regulated genes in G‐LLC or PBS treated BMDCs. (H) The volcano plot showing the differentially regulated genes in IR‐G‐LLC or G‐LLC treated BMDCs. (I) Heatmap of the differentially expressed genes in PBS, G‐LLC, and IR‐G‐LLC treated BMDCs. (J) GO analysis of signaling pathways enrichment in IR‐G‐LLC treated BMDCs compared to G‐LLC treated BMDCs. **p < 0.01 and ***p < 0.001.

It has been reported that irradiated tumor cells can activate DCs and macrophages.^[^ ^30^ ^]^ We tested the immune‐stimulating ability of the GM‐CSF overexpressed irradiated tumor cell debris. Bone marrow‐derived dendritic cells (BMDCs) and Bone marrow‐derived macrophages (BMDMs), generated from bone marrow of C57BL/6 mice, were treated with PBS, Lewis cell debris (LLC), GM‐CSF overexpressed Lewis cell debris (G‐LLC), irradiated GM‐CSF overexpressed Lewis cell debris (IR‐G‐LLC) for 24 h, and CD86 expression on BMDCs and BMDMs was evaluated by flow cytometry. As shown in Figure 2A,B, compared to PBS, LLC, G‐LLC and IR‐G‐LLC induced higher expression of CD86 in BMDCs, where IR‐G‐LLC exhibited the highest potential. Similar results were observed in BMDMs where IR‐G‐LLC induced the highest level of CD86 (Figure 2C,D). We then examined the immune‐activating ability of HA@RTD hydrogel on BMDCs and BMDMs and found that the hydrogel formation did not affect activation ability of IR‐G‐LLC (Figure 2E,F, Figure S2A,B). Hence, these results indicate that HA@RTD hydrogel can activate BMDCs and BMDMs.

RNA sequencing (RNA‐seq) of untreated, G‐LLC, or IR‐G‐LLC treated BMDCs indicated that IR‐G‐LLC could further promote BMDCs activation compared with G‐LLC. Compared to G‐LLC treatment, IR‐G‐LLC treatment further upregulated the expression of Nos2, IL‐1β, CXCL2, CXCL3, CCL3, CCL5, which are markers for DCs activation and chemokines for T cells recruitment (Figure 2G–I). We also validated some of the sequencing data by RT‐qPCR (Figure S2C). KEGG analysis revealed that TNF signaling and MAPK signaling pathways were significantly upregulated in IR‐G‐LLC treated BMDCs (Figure 2J). GSEA analysis showed that immune activation pathways were activated in IR‐G‐LLC treated BMDCs (Figure S2D). Collectively, IR‐G‐LLC could activate immune responses and retained the ability when self‐assembly into hydrogel.

### 2.3 In vivo preventive and therapeutic effect of HA@RTD hydrogel vaccine

**FIGURE 3:**
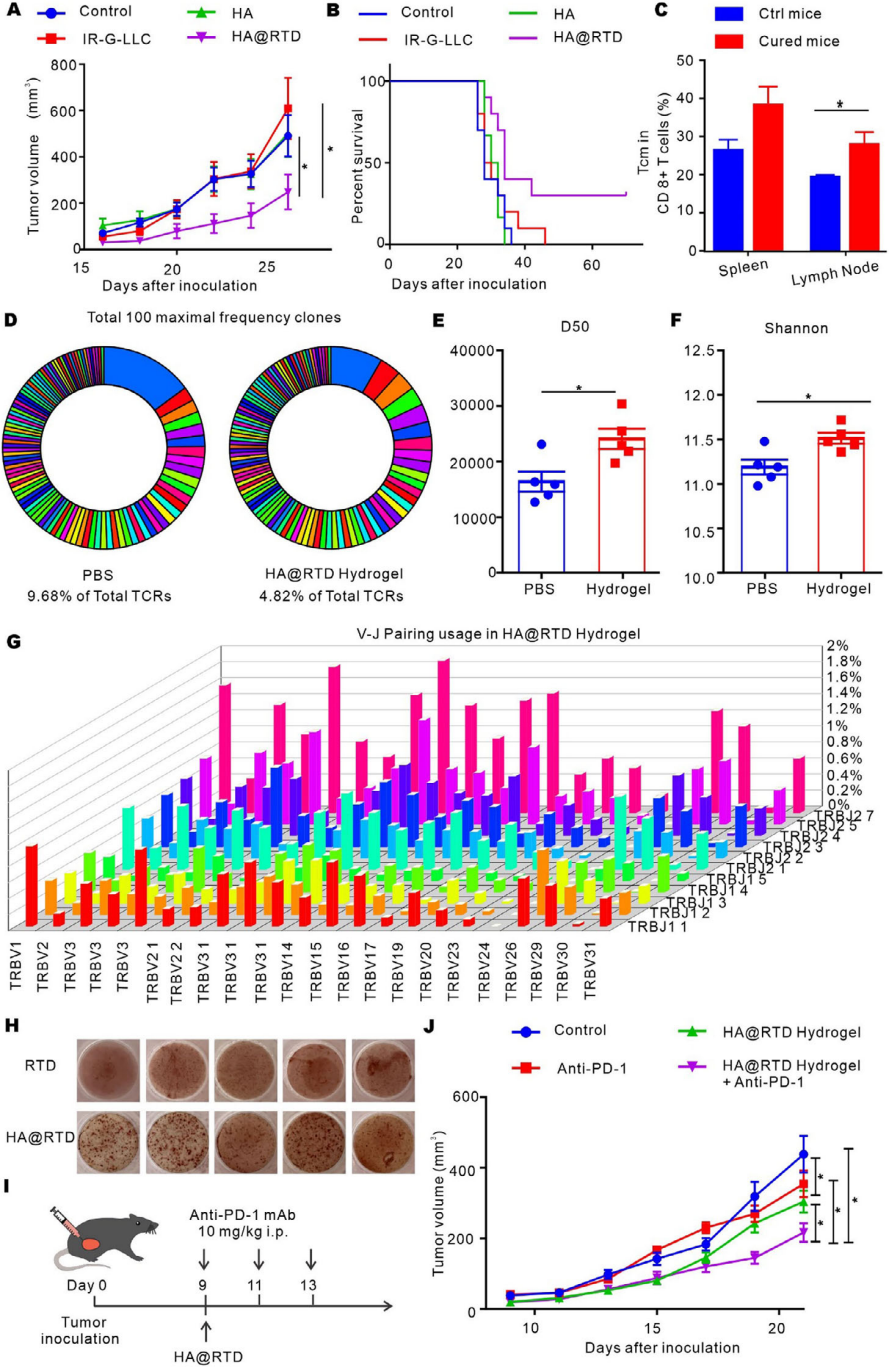
HA@RTD hydrogel inhibited tumor growth and increased TCR diversity. (A) Tumor volume curves and (B) survival curves of mice after pre‐treated with PBS, HA, IR‐G‐LLC, and HA@RTD (*n* = 8–10 per group). (C) Flow cytometry analysis of the CD8^+^ memory T cells in spleen and lymph nodes. (D) Representative images of total 100 maximum frequency clones in PBS and HA@RTD treated mice. (E) D50 value of T cells in PBS and HA@RTD treated mice. (F) Shannon index of T cells in PBS and HA@RTD treated mice. (G) Representative image of V–J paring usage of spleen T cells in HA@RTD treated mice. (H) Images of ELISpot result. (I,J) Tumor growth curves of Lewis cell subcutaneous model in the indicated groups (*n* = 9). *p < 0.05.

We next examined the preventive effect of HA@RTD hydrogel in C57BL/6 mice. We tested a subcutaneous tumor model in mice 10 days after intradermal injection of PBS, IR‐G‐LLC, HA, and HA@RTD hydrogel, respectively. Compared to other groups, HA@RTD hydrogel effectively inhibited tumor growth and prolonged the survival, and three mice were free of tumor for over two months (Figure 3A,B). To further study if HA@RTD hydrogel promoted the formation of memory immune responses, we measured the ratio of CD8^+^ Tcm (CD3^+^CD8^+^CD44^+^CD62L^high^) in spleen and inguinal lymph node of the 3 tumor free mice after HA@RTD hydrogel treatment.^[^ ^31^ ^]^ Compared with the same aged mice, we found that the CD8^+^ Tcm ratio was significantly increased in the HA@RTD hydrogel cured group, demonstrating the formation of memory immune responses (Figure 3C). T cells are the main executors of anti‐tumor immunity and are the most important cells for tumor vaccine evaluation. We explored the T cell clonotypes and diversity by characterizing the tumor TCRβ repertoires with spleen of HA@RTD hydrogel treated mice and control mice. The TCRβ CDR3 sequences were firstly amplified by several V‐primers and J‐primers, and then were subjected to high‐throughput sequencing. The data were mapped to the international ImMunoGeneTics (IMGT) database to obtain V, D and J fragment, rearrangement and CDR3 sequences.^[^ ^32^, ^33^ ^]^ As shown in Figure 3D–F, we demonstrated that HA@RTD hydrogel treatment increased the CDR3 diversity with higher D50 and Shannon index. All the TCR rearrangement sequences in the sample are arranged according to the proportion in the sample from high to low, and then the proportion of TCR sequences is added in this order. When the addition ratio reaches half of the sample, the number of TCR rearrangement sequences added is D50. The higher the D50 value, the higher the TCR diversity of the sample. The TCR sequencing showed a higher mutation in both V, D and J segments, which means the HA@RTD hydrogel induced a high diversity of T cells. The V–J pairing usage in control and HA@RTD hydrogel group was shown in Figure S3A and Figure 3G. To evaluate the vaccine‐induced specific immunity, we performed enzyme‐linked immune absorbent spot (ELISpot) assay for IFN‐γ. As shown in Figure 3H, the spleen cells from HA@RTD vaccinated mice showed more spots compared to the RTD group, indicating more IFN‐γ secreting cells in HA@RTD group.

To evaluate the in vivo therapeutic effect, after subcutaneous LLC tumors reached 50 mm^3^, mice were randomly divided into four treatment groups and treated once with PBS, IR‐G‐LLC, HA, and HA@RTD hydrogel, respectively. As shown in Figure S3B, HA@RTD hydrogel significantly inhibited the tumor growth. When combined with anti‐PD‐1 mAb, HA@RTD enhanced the anti‐tumor effect of anti‐PD‐1 mAb and cured two mice in nine (Figure 3I,J and Figure S3C). Taken together, these results indicate that HA@RTD hydrogel exhibited preventive and therapeutic anti‐tumor effect in vivo.

### 2.4 HA@RTD hydrogel enhanced immune activation

**FIGURE 4:**
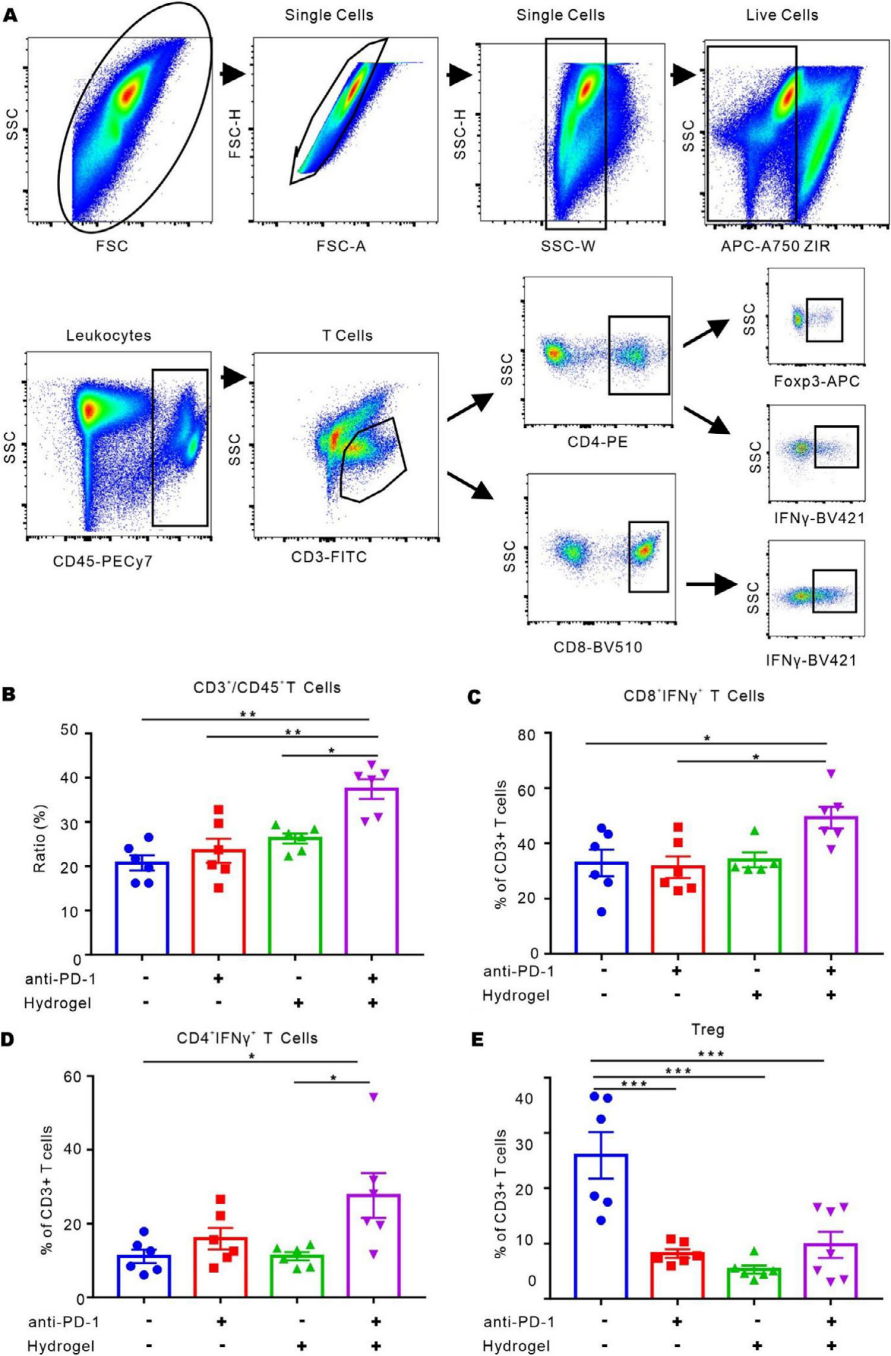
HA@RTD hydrogel vaccine activated anti‐tumor immunity. (A) Flow cytometry gating strategy for the detection of CTL, Th1, and Treg cells. (B–D) Flow cytometry analysis of CTL, Th1, and Treg in TME. (B) The ratio of CD3^+^ T cells to CD45^+^ cells. (C) Proportions of CTL, (D) proportions of Th1, (E) proportions of Treg among CD3^+^ T cells. *p < 0.05, **p < 0.01 and ***p < 0.001.

To evaluate the effects of HA@RTD hydrogel on the T cells in tumor microenvironment (TME), we profiled the proportion of cytotoxic T lymphocytes (CTLs), T helper type 1 (Th1) cells, and regulatory T (Treg) cells in TME by flow cytometry. Mice bearing subcutaneous LLC tumors were treated with PBS, HA@RTD hydrogel, anti‐PD‐1 mAb, or HA@RTD hydrogel combined with anti‐PD‐1 mAb. The percentages of tumor‐infiltrating T cells were analyzed at 10 days after treatment, and the gating strategy and representative diagrams for CTLs (ZIR^−^CD45^+^CD3^+^CD8^+^IFN‐γ^+^), Th1 cells (ZIR^−^CD45^+^CD3^+^CD4^+^IFN‐γ^+^), and Tregs (ZIR^−^CD45^+^CD3^+^CD4^+^Foxp3^+^) were shown in Figure 4A.^[^ ^34^, ^35^ ^]^ Our results showed that the combination group had the highest CD3^+^/CD45^+^ T‐cell ratio, indicating more T cells infiltration (Figure 4B). More specifically, HA@RTD hydrogel plus anti‐PD‐1 mAb induced significant higher CTLs infiltration compared to anti‐PD‐1 mAb group (Figure 4C), and induced higher Th1 cells compared to HA@RTD hydrogel alone (Figure 4D), while significantly decreased the portion of Treg cells (Figure 4E). In our model, we noticed that anti‐PD‐1 alone decreased the portion of Treg cells. PD‐1 also expressed on Treg cells and the antibody may induce antibody dependent cell‐mediated cytotoxicity, which may explain the decrease of Treg cells.^[^ ^36^ ^]^ The CD4^+^/CD8^+^ T‐cell ratio exhibited no difference among groups (Figure S4). These results suggest that the HA@RTD hydrogel plus anti‐PD‐1 mAb promoted the infiltration of CTLs and Th1 cells, but decreased Tregs cells, in the TME.

### 2.5 HA@RTD hydrogel induced immune memory

**FIGURE 5:**
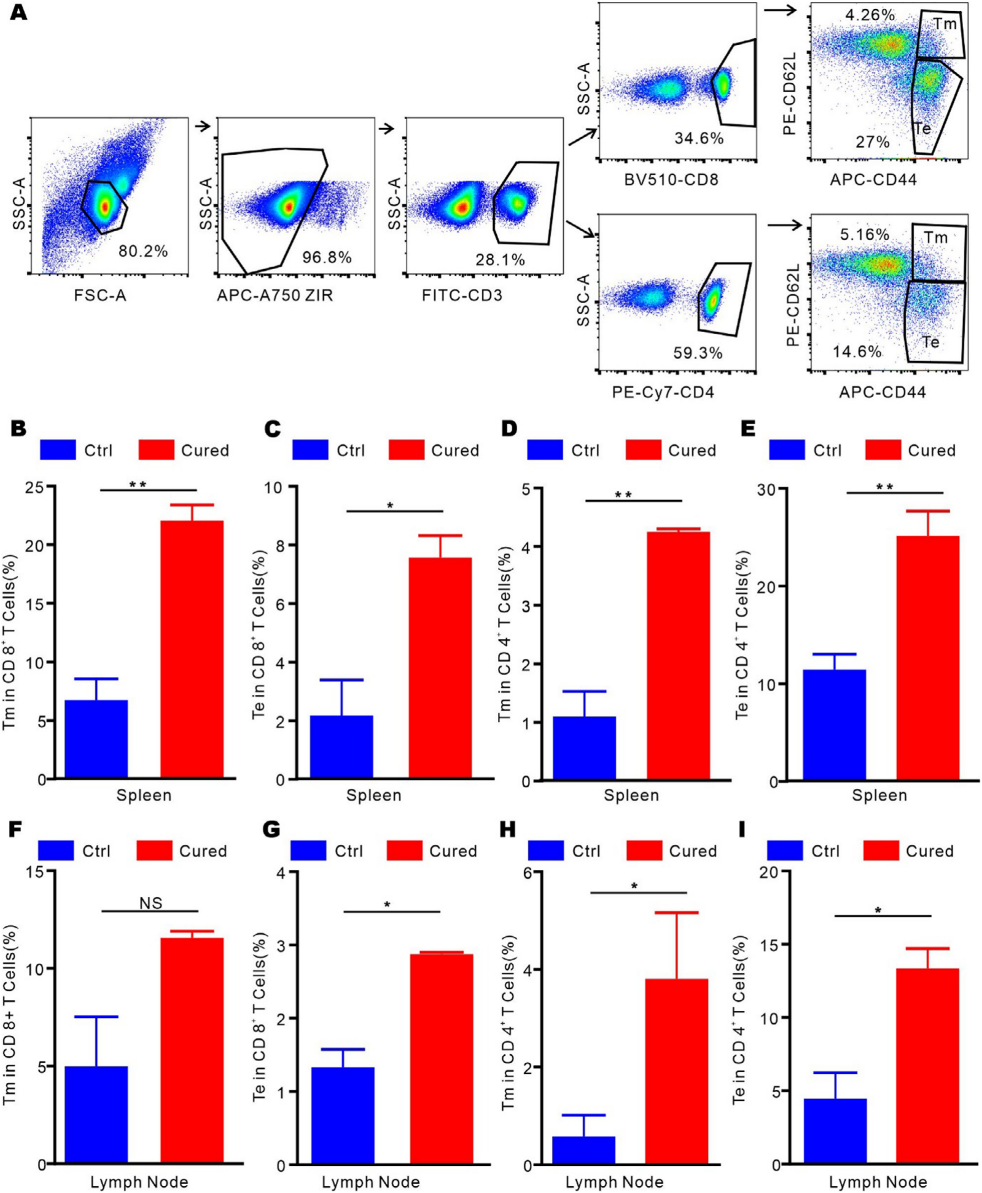
HA@RTD hydrogel vaccine induced immune memory. (A) Gating strategy for memory T cells. (B–E) Statistical analysis of memory T cells in spleen. (F–I) Statistical analysis of memory T cells in lymph nodes. *p < 0.05, **p < 0.01.

Since HA@RTD hydrogel plus anti‐PD‐1 mAb cured two mice in nine, we then studied whether memory immune responses were induced in the cured mice. Sixty days after the mice cured, we measured the ratio of CD8^+^ Tem, CD8^+^ Tcm and CD4^+^ Tem, CD4^+^ Tcm in spleen and inguinal lymph node of cured mice and the control mice (in same age). Compared to the control mice, we found that the population of CD8^+^ Tem, CD8^+^ Tcm, CD4^+^ Tem, and CD4^+^ Tcm were significantly increased in both spleen and inguinal lymph node of the cured group, suggesting the induction of memory immune responses (Figure 5). Collectively, these results indicate that the combination of HA@RTD hydrogel with PD‐1 blockade induced immune memory responses, thus conferring a long‐term anti‐tumor effect.

### 2.6 Biocompatibility of HA@RTD hydrogel

**FIGURE 6:**
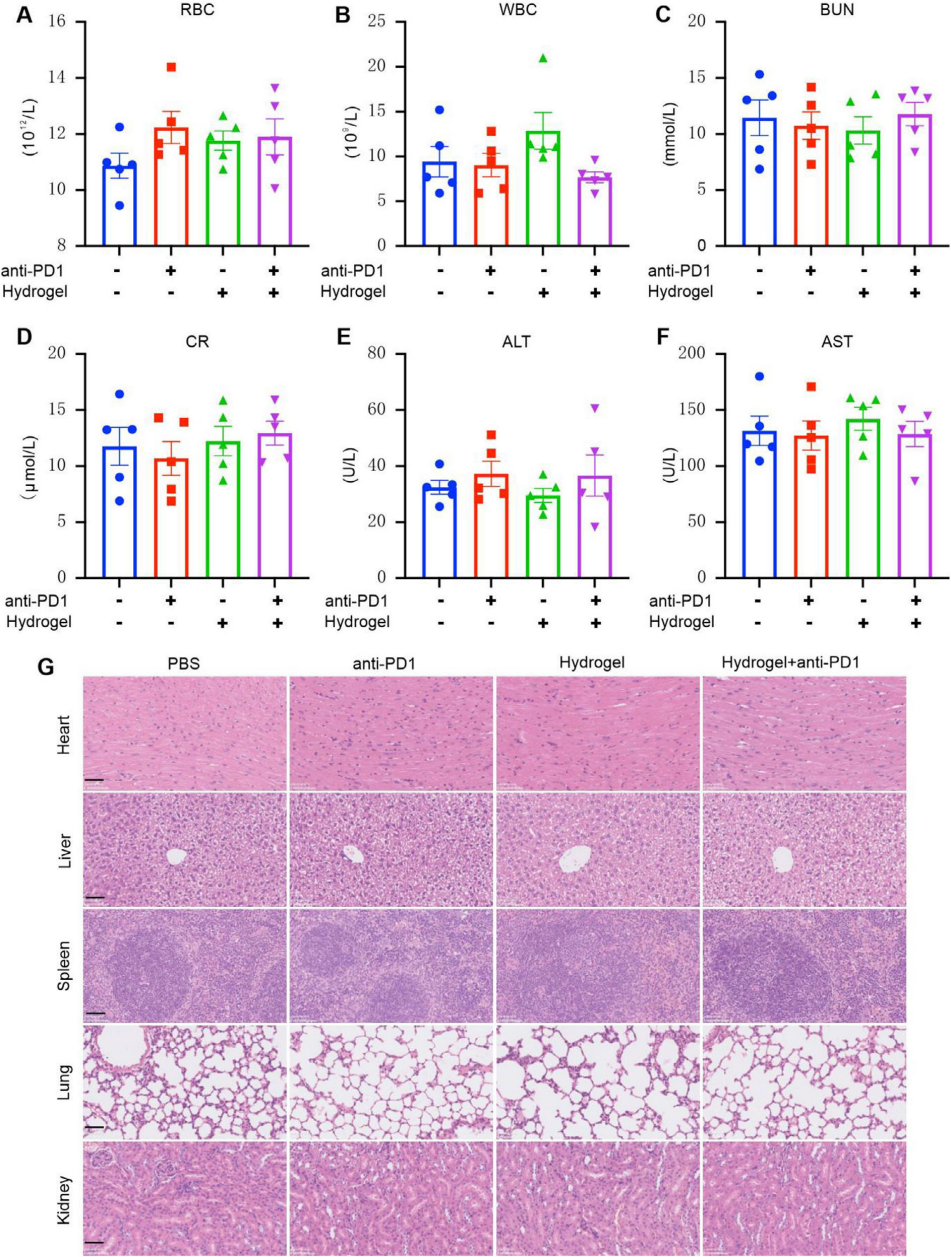
Biocompatibility of HA@RTD hydrogel. (A–F) The count of Red blood cell (RBC), White blood cell (WBC), and the concentration of Blood urea nitrogen (BUN), creatinine (CR), Alanine Transaminase (ALT) and Aspartate Transaminase (AST) in the indicated groups. (G) Representative histological examinations of the main organs with hematoxylin and eosin (H&E) staining in the indicated group. Scale bar = 100 µm.

To assess the biocompatibility of HA@RTD hydrogel, blood and tissue were collected from the mice at fifteen days post‐treatment for biochemical analysis and hematoxylin‐eosin (H&E) staining. No differences were observed in the number of red blood cells (RBC), white blood cells (WBC), blood urea nitrogen (BUN), creatinine (CR), alanine transaminase (ALT), and aspartate aminotransferase (AST) among the four groups (Figure 6A–F). Also, histopathological examination of heart, liver, spleen, lung, and kidney showed that these major organs were not affected by HA@RTD hydrogel treatment or combined treatment (Figure 6G). Therefore, these data suggest that the HA@RTD hydrogel is safe for in vivo treatment.

## 3 CONCLUSION

Insufficient activation of anti‐tumor T cells has been a major factor limiting vaccine efficacy. Hence, we constructed a novel HA@RTD hydrogel with advantages in three aspects: (1) Irradiated tumor cell debris not only contains a broad spectrum of tumor antigens but also personalized antigens for the tumor, which can promote the TCR diversity and enhance the anti‐tumor effect for the heterogeneous tumor cells; (2) Overexpressing GM‐CSF on the membrane helps activate the DCs and have the adjuvant effect without the toxicity. (3) HA with high biocompatibility improved the persistence of antigen stimulation and induce a more durable immune response. Also, we achieved tumor inhibition by just a single injection. Taken together, this novel tumor vaccine significantly activates anti‐tumor immunity and promote the infiltration of T cells, which provides a potentially transformable individualized tumor vaccine. There are still limitations and areas of improvement for this vaccine. The therapeutic effect of the vaccines in established murine tumors were not sufficient for curative responses, even when combined with anti‐PD‐1 mAb. More efforts are needed to amplify the anti‐tumor immunity. As for the translational potential for this vaccine approach, obtaining primary tumor cells from patients and expanding them ex vivo is feasible, making it possible for the vaccine approaches like this one to move into clinic testing in the future.

## 4 EXPERIMENTAL SECTION

### 4.1 Preparation of HA‐NHS

Hyaluronic acid was dissolved in deionized water at 2 wt%. The 120 µL of 2 wt% alginate solution was mixed with 5 mg of 1‐ethyl‐3‐(−3‐dimethylamino‐propyl) carbodiimide (EDC) (Thermo Scientific) and 3 mg of *N*‐hydroxysulfosuccinimide sodium salt (sulfo‐NHS) (ACROS Organics). The water layer was washed with dichloromethane and dialyzed in ice water for 12 h using dialysis bag, followed by freeze‐dry to get the HA‐NHS.

### 4.2 Construction of HA@RTD hydrogel

GM‐CSF overexpressed tumor cells grown in cell culture dishes were treated with 20 Gray radiation by 6 MV X‐rays (600 MU min^−1^; Trilogy System Linear Accelerator, Varian Medical Systems). After 96 h, the tumor cells were collected, washed by PBS, and subjected to ultrasonication (200 W, 5 s working time and 10 s resting time, and last for 10 min), followed by centrifugation at 10,000 × *g* for 30 min to harvest the debris of radiated tumor cells. The debris of 10 million tumor cells was resuspended with 1 mL PBS and mix with 0.2 mg HA‐NHS to construct the HA@RTD hydrogel, which was stored at 4°C until further use.

### 4.3 Scanning electron microscope

The morphology of HA‐NHS and HA@RTD hydrogel was imaged by SEM (FESEM, China). The samples were precooled at −80°C for 1 hour and freeze‐dried in a low‐temperature vacuum dryer (YW‐10A) for 24 h. The freeze‐dried samples were pasted on the conductive adhesive and the surface of the samples was coated with gold film by ion sputtering instrument (SBC‐12). Images were obtained by SEM.

### 4.4 Rheological analysis

Rheometer (DHR2, TA company) was used to determine the rheological properties of the HA@RTD hydrogel, under following parameters: strain force 0.1%, angular frequency from 0.1 to 100 rad s^−1^.

### 4.5 Cell lines and cell culture

Murine lung carcinoma cells LLC was purchased from the American Tissue Culture Collection (ATCC). LLC‐GM‐CSF cells stably expressing GM‐CSF were generated by lentivirus transduction. Cells were maintained in Dulbecco's Modified Eagle Medium (DMEM) containing 10% fetal Bovine Serum (FBS) and 1% penicillin and streptomycin at 37°C with 5% CO_2_.

### 4.6 DiD and Cy5 releasing in vitro and in vivo

DiD release from the HA hydrogel was detected in vitro using MicroplateReader, with excitation wavelength of 644 nm and emission wavelength of 665 nm. The excitation wavelength for Cy5 is 654 nm and emission wavelength is 670 nm. The 0.5 mL HA (containing 0.1 mg DiD and 0.2 mg HA in 0.9% NaCl solution) was added to a 1.5 mL eppendorf tube and 0.5 mL 0.9% NaCl buffer with or without 5 unit mL^−1^ proteinase K was added at 37°C. At indicated time points, 10 µL sample was mixed with 190 µL 0.9% NaCl buffer, which was stored in −20°C and measured at the same time. For in vivo detection, 100 µL HA‐DiD (1 µm) or free DiD (1 µm), RTD‐Cy5 (0.5 µm) or HA@RTD‐Cy5 (0.5 µm) was subcutaneously injected in mice and the signal was detected by the ChemiDoc MP Imaging System at different time points.

### 4.7 Generation of BMDCs and BMDMs

Bone marrow cells were collected from the femurs of 6−8‐week‐old C57BL/6 mice. To generate BMDCs, bone marrow cells were differentiated in RPMI 1640 complete medium supplemented with 20 ng mL^−1^ GM‐CSF and the complete medium was refreshed every two days, BMDCs were harvested on the seventh day. To generate BMDMs, bone marrow cells were differentiated in RPMI 1640 complete medium supplemented with 20 ng mL^−1^ M‐CSF that was refreshed every two days. On the fifth day, IL‐4 (20 ng mL^−1^, PeproTech) was supplemented in medium to generate M2 type macrophages (BMDM‐M2), which were got on the seventh day.

### 4.8 Flow cytometry analysis

After different treatments, BMDMs and BMDCs were collected and stained with anti‐CD80 (PE) and anti‐CD86 (APC) antibodies for 30 min on ice. After wash with PBS for three times, cells were resuspended with PBS and analyzed by flow cytometry.

### 4.9 RNA‐seq

Total RNA was extracted from BMDCs treated with PBS, G‐LLC lysis, or IR‐G‐LLC lysis for 24 h, which were sent to BGI (Shenzhen, China) for sequencing. Total RNAs were extracted from samples using TRIzol. Experimental process includes sample testing, mRNA separation, mRNA interruption, cDNA synthesis, PCR, library detection, cyclization, and computer sequencing.

### 4.10 T‐cell receptor (TCR)‐seq

The HA@RTD hydrogel or PBS was injected intradermally into the 6−8‐week‐old C57BL/6 mice. Two weeks later, the spleen samples were collected and sent to the Seqhealth Technology Co., Ltd., Wuhan, China (http://www.seqhealth.cn) for sequencing and analyzing.

### 4.11 ELISpot

The HA@RTD hydrogel or the RTD were used to vaccine 10 mice every 14 day. The mice were sacrificed to get the spleen cells 14 days later after the last vaccination. Culture the spleen cells in medium with IL‐2 and dilute them to the same concentration. Add the cells to the ELISpot kit (mouse IFN‐γ Precoated ELISPOT Kit, 2210005) and stimulate them with RTD or HA@RTD. Test the spot production following the kit protocol.

### 4.12 Animal studies

Male C57BL/6 mice were purchased from Liaoning Changsheng Biotechnology Co., Ltd. Animals were raised in a specific pathogen‐free barrier facility in the Animal Center of the Huazhong University of Science and Technology (HUST), Wuhan, China. All animal studies were performed in compliance with protocols that had been approved by the Hubei Provincial Animal Care and Use Committee, in accordance with the experimental guidelines of the Animal Experimentation Ethics Committee of the HUST (Wuhan, China). The IACUC Number was 3426.

To evaluate preventive effect, PBS, IR‐T‐LLC, HA, or HA@RTD hydrogel was injected intradermally at the base of the tail and 10 days later to build subcutaneous mouse model. The length (*L*) and width (*W*) of tumors were measured and the tumor volume (*V*) was calculated using the formula *V = (L* × *W* ^2^)/2. The tumor volume was recorded every two days. To evaluate therapeutic effect, one million LLC cells were injected subcutaneously into the right flanks of mice. Seven days after the injection, mice were randomly divided into four groups and received a single intradermal administration of PBS (50 µL), IR‐G‐LLC (100 µg, 50 µL), HA (50 µL), and HA@RTD hydrogel (100 µg, 50 µL), respectively.

### 4.13 Quantification and statistical analysis

Statistical analysis was performed using GraphPad Prism 6.0 software. One‐way ANOVA with Tukey's multiple comparisons test was used to compare three or more groups. A two‐tailed unpaired *t*‐test was used to compare two groups. *p*‐value < 0.05 was considered statistically significant. Data are presented as mean ± standard error of the mean (SEM). *p < 0.05, **p < 0.01, ***p < 0.001, NS: not significant.

## AUTHOR CONTRIBUTIONS

Yajie Sun, Yan Hu, Yuanyuan Geng: Investigation, Methodology, Writing‐Original draft preparation. Chao Wan: Investigation, Validation. Yang Liu: Software, Validation. Yifei Liao and Xiujuan Shi: Review and editing. Jonathan F. Lovell: Formal analysis, Resources. Kunyu Yang, Honglin Jin: Supervision, Resources, Funding acquisition, Writing—Reviewing and Editing.

## CONFLICT OF INTEREST STATEMENT

The authors declare no conflicts of interest. Jonathan F. Lovell is a member of the *Exploration* editorial board.

## Supporting information

Supporting Information

## Data Availability

The data that support the findings of this study are available from the corresponding author upon reasonable request.
