# Supplementary material for: A self‐assembled, genetically engineered, irradiated tumor cell debris vaccine
Source: Exploration (Beijing). 2024 Mar 6;4(5):20220170. doi: 10.1002/EXP.20220170 (PMC11491297; doi:10.1002/EXP.20220170)
Supplement: Supplementary file 1 — Supporting Information [file EXP2-4-20220170-s001.docx]

**Supplementary Figures:**


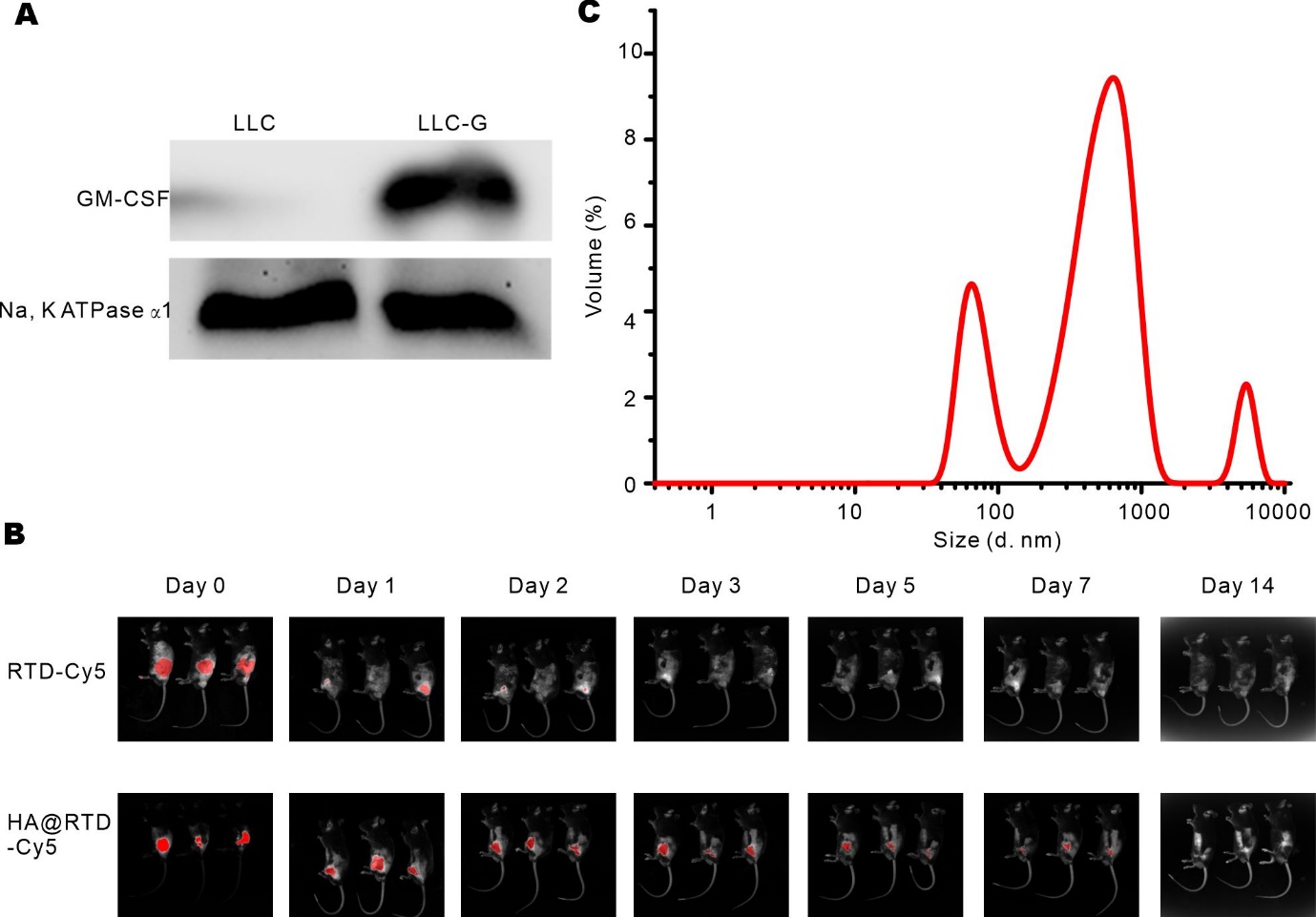


**Figure S1. The characterization of G-LLC and tumor debris.**

(A) image of western blot of GM-CSF. (B) The Cy5 imaging of the distribution of the RTD-Cy5 and HA@RTD-Cy5 at indicated time points *in vivo*. (C) Representative size distribution of irradiated tumor cell debris.


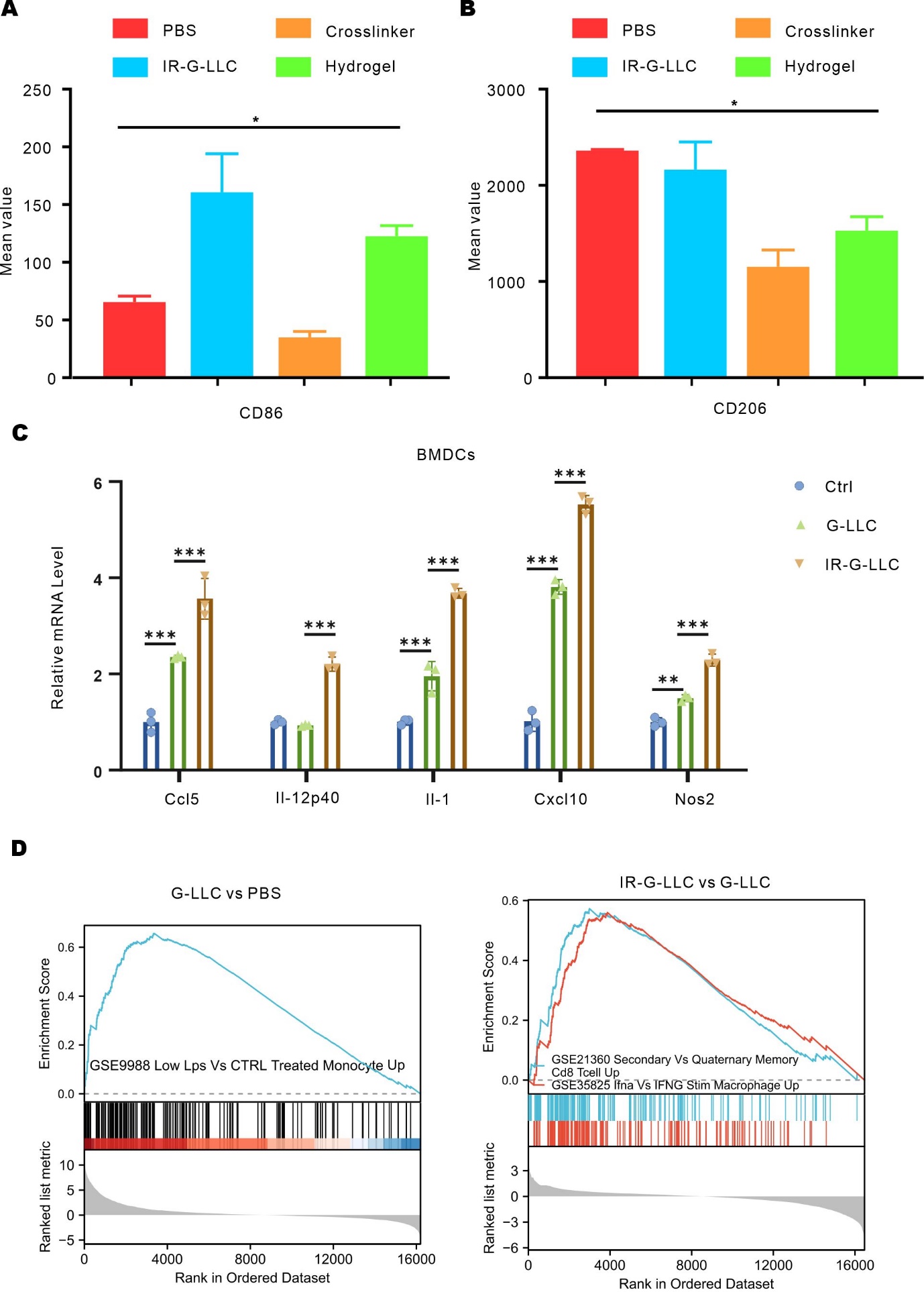


**Figure S2.** **HA@RTD hydrogel vaccine activated BMDMs.**

(A-B) CD86 (A) and CD206 (B) expression in BMDMs treated with PBS, IR-G-LLC, HA, OR HA@RTD. (C) RT-qPCR data for some of the gene’s validation. (D) GSEA analysis of immune related pathways.


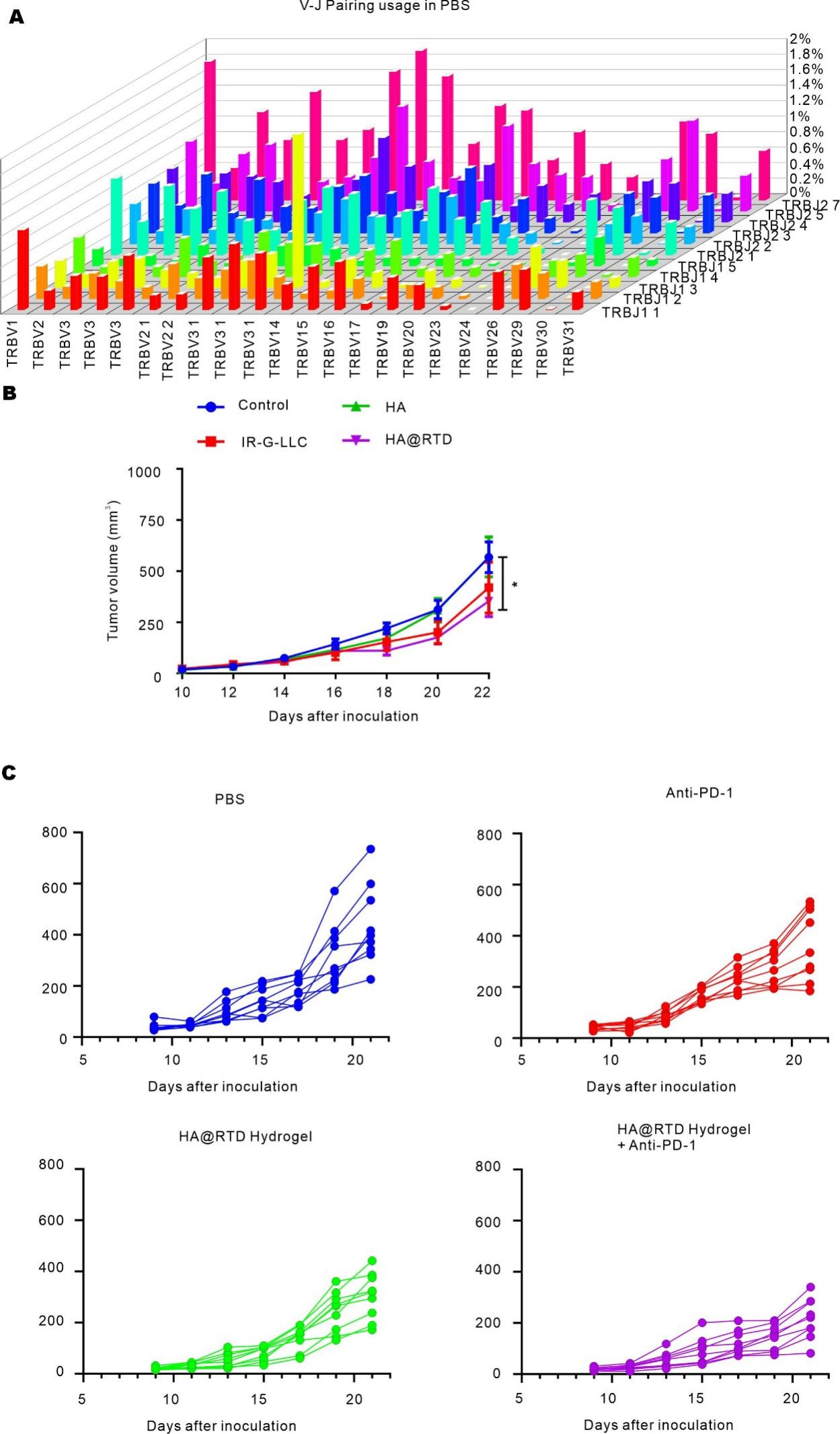


**Figure S3.** **HA@RTD hydrogel vaccine inhibited tumor growth.**

(A) Representative image of V-J paring usage of spleen T cells in control mice. (B) Tumor growth curves of LLC cell subcutaneous model in the indicated groups (n = 8-10). (C) Individual tumor growth curves for mice in Figure 3I.


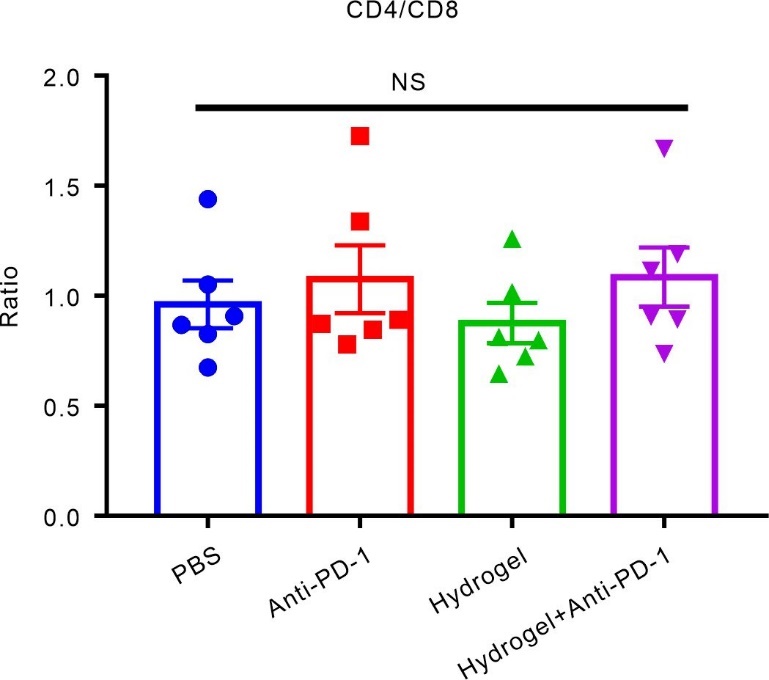


**Figure S4.** **Flow cytometry analysis of the CD4/CD8 ratio in the indicated groups.**
